# N^6^-methyladenosine modification of SLC38A7 promotes cell migration, invasion, oxidative phosphorylation, and mitochondrial function in gastric cancer

**DOI:** 10.1016/j.jbc.2024.107843

**Published:** 2024-09-30

**Authors:** Yi Hua, Wei-Jun Hua, Cun-Cheng Feng, Qiu-Wei Zhu

**Affiliations:** Department of Gastrointestinal Surgery, The Affiliated Changzhou No.2 People’s Hospital of Nanjing Medical University, Changzhou, China

**Keywords:** gastric cancer, solute carrier family, m^6^A methylation, metastasis, oxidative phosphorylation

## Abstract

Solute carrier (SLC) 38 family, responsible for trans-membrane transport of neutral amino acids, plays a role in the proliferation, invasion, and metastasis of cancer cells, but its role in gastric cancer (GC) progression remains unclear. This study aimed to explore the biological effects of SLC38A7 and its regulatory mechanisms in GC. RNA expression data, tumor tissue specimens, and GC cell lines were used for bioinformatics and experimental analyses. Cell Counting Kit-8 assay, wound healing assay, and Transwell invasion assay were used to evaluate cell viability, migration, and invasion, respectively. Oxidative phosphorylation, mitochondrial membrane potential, and expression of the critical proteins in the mitochondrial respiratory chain were assayed using extracellular flux analysis, flow cytometry, and Western blot, respectively. RNA immunoprecipitation assay was used to explore the mechanisms of N6-methyladenosine (m^6^A) methylation. SLC38A7 was upregulated in GC tissue and cell lines. *SLC38A7* silencing suppressed cell viability, migration, invasion, oxidative phosphorylation, and mitochondrial function in cancer cells. *SLC38A7* overexpression had the opposite biological effects. Interactions between SLC38A7 and methyltransferase like 3 (METTL3) or insulin-like growth factor 2 mRNA-binding protein 2 (IGF2BP2) were detected. *SLC38A7* mRNA stability was maintained by METTL3–IGF2BP2 axis in an m^6^A-dependent manner. Our results suggest that SLC38A7, stabilized by METTL3 and IGF2BP2-mediated m^6^A methylation, enhances cell viability, migration, invasion, oxidative phosphorylation, and mitochondrial function in GC, highlighting its role as a potential therapeutic target for GC.

Gastric cancer (GC) is the fourth leading cause of global cancer-related mortality (1). It is a highly heterogeneous disease with respect to molecular characteristics and phenotype (2). Cancer metabolism is characterized by typical alterations, such as high glutamine dependence, increased glucose intake, and strengthened glycolysis (3). Glutamine plays an important role in anabolic metabolism by supplying nitrogen to purines and pyrimidines (4) and serves as a fuel which produces metabolic intermediates through the tricarboxylic acid cycle, thereby acting as a footstone for lipids, proteins, and nucleic acids, which are vital for anabolic development (5, 6). Mitochondria are the main cellular power generators, providing most of their energy in the form of ATP, which fuels function through oxidative phosphorylation. A previous study demonstrated that glutamine supplementation yielded a higher oxygen consumption rate (OCR) and increased ATP content (7), suggesting that glutamine serves as a major source to fuel oxidative phosphorylation. Inhibiting glutamine metabolism is now recognized as a promising anticancer therapeutic strategy (8). However, it remains unknown whether glutamine can modulate oxidative phosphorylation and mitochondrial function that favors GC progression.

Solute carrier (SLC) 38 family, namely the sodium-coupled neutral amino acid transporter (SNAT) family, which comprises 11 members, is responsible for the transport of neutral amino acids across biological membranes, with glutamine being the most common substrate (9). SLC38A7 encodes SNAT7 which is a lysosomal glutamine transporter highly selective for the transport of glutamine and asparagine from lysosomal lumen into cytoplasm (10). SNAT7 links to the mammalian target of rapamycin complex 1, thus playing a regulatory role in the growth and metabolism of pancreatic cancer cells (11). High SLC38A7 expression predicts poor prognosis and is a potential therapeutic target in lung squamous cell carcinoma (12). However, the specific roles of SLC38A7 in GC are not well characterized. A recent study suggests upregulation of SLC38A2 in GC, which promotes proliferation, invasion, and migration of GC cells, leading to poor prognosis (13). Therefore, we speculate that SLC38A7 may affect the proliferation, invasion, and migration of GC cells.

In the present study, we used lentivirus-mediated *SLC38A7* silencing and overexpression models to investigate the effects of SLC38A7 on GC cell viability, invasion, migration, oxidative phosphorylation, and mitochondrial function. Furthermore, the underlying regulatory mechanisms were also explored. Our findings may provide insights into the biological activities of SLC38A7 in carcinogenesis and tumor progression.

## Results

### SLC38A7 was upregulated in GC patients and predicted poor prognosis

**Figure 1:**
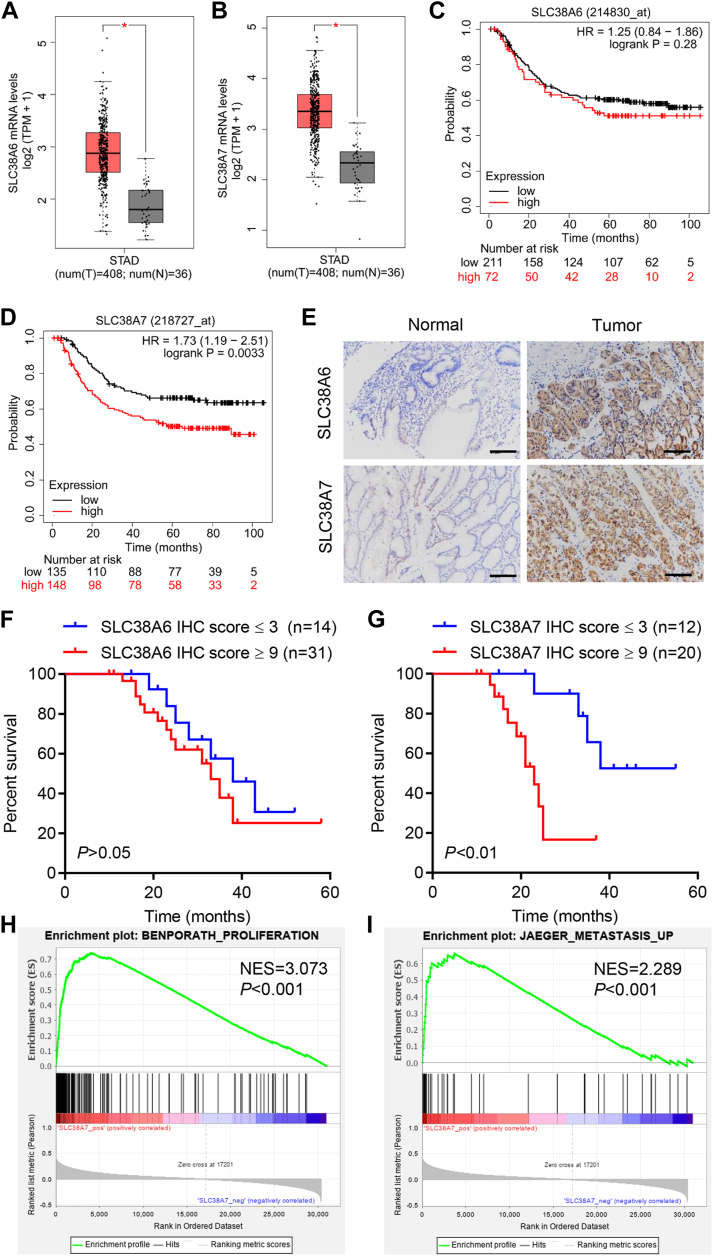
**SLC38A7 is elevated in GC and indicates a poor prognosis.***A* and *B*, analysis of mRNA expression data of *SLC38A6* and *SLC38A7* in GC patients from TCGA using GEPIA. *C* and *D*, survival outcomes of GC patients in the GSE62254 database. *E*, analysis of SLC38A6 and SLC38A7 protein in GC tissue microarray by IHC staining (scale bar represents 100 μm). *F* and *G*, survival analysis of GC patients based on tissue microarray using the Kaplan-Meier method. *H* and *I*, gene set enrichment analysis of *SLC38A7* associated with proliferation and metastasis. ∗*p* < 0.05 *versus* normal.

The expression of SLC38 family members including SLC38A1-11 in GC patients was analyzed in The Cancer Genome Atlas (TCGA) data using the Gene Expression Profiling Interactive Analysis (GEPIA) platform. As shown in Fig. 1, *A* and *B*, *SLC38A6* and *SLC38A7* mRNA levels were markedly increased in GC samples compared to control samples. However, there was no significant difference in the expression of other SLC38 family members between GC and control samples (data not shown). Furthermore, there was no significant difference in the overall survival between patients with high and low *SLC38A6* mRNA expression in the GSE62254 database (Fig. 1*C*, *p* > 0.05). However, patients with low *SLC38A7* expression showed significantly longer survival than patients with high *SLC38A7* mRNA expression (Fig. 1*D*, *p* < 0.01). SLC38A6 and SLC38A7 expressions in tumor tissue sections were higher than those in normal tissue sections (Fig. 1*E*). There was no significant difference in overall survival time between patients with SLC38A6 immunohistochemical (IHC) scores ≤ 3 and ≥ 9 (Fig. 1*F*, *p* > 0.05). However, patients with SLC38A7 IHC score ≤ 3 showed significantly longer survival than patients with SLC38A7 IHC score ≥ 9 (Fig. 1*G*, *p* < 0.01), suggesting a prognostic relevance of SLC38A7 expression in GC patients. As shown in Table 1, SLC38A7 expression also showed a significant association with tumor size (*p* = 0.028) and TNM stage (*p* = 0.027). We further performed gene set enrichment analysis to investigate the potential signal pathways affected by SLC38A7 and found a positive correlation of SLC38A7 with proliferation and metastasis pathways (Fig. 1, *H* and *I*). SLC38A7 mRNA and protein levels in human gastric cancer cell lines (AGS, HGC27, MKN45, and NCIN87) were higher than those in normal human gastric epithelium cells (GES-1), with the highest expression detected in AGS cells and the lowest expression detected in HGC27 cells (Fig. S1*A*). These two cell lines were therefore used for subsequent experiments.

**Table 1:** Relationship between SLC38A7 expression and clinicopathological features of patients with GC

Clinicopathological features	SLC38A7		*p* value
	IHC score ≤ 3 (n = 12)	IHC score ≥ 9 (n = 20)	
Gender			0.234
Male (n = 15)	4	11	
Female (n = 17)	8	9	
Age (years)			0.358
≤58 (n = 14)	4	10	
>58 (n = 18)	8	10	
Location			0.636
Cardia (n = 13)	6	7	
Corpus (n = 11)	3	8	
Antrum (n = 8)	3	5	
Tumor size (cm)			0.028
≤3 (n = 16)	9	7	
>3 (n = 16)	3	13	
TNM stage			0.027
I (n = 7)	5	2	
II (n = 9)	5	4	
III (n = 13)	2	11	
IV (n = 3)	0	3	

Differences between groups were done by the Chi-square test.

### *SLC38A7* knockdown inhibited cell viability, migration, and invasion in AGS cells

**Figure 2:**
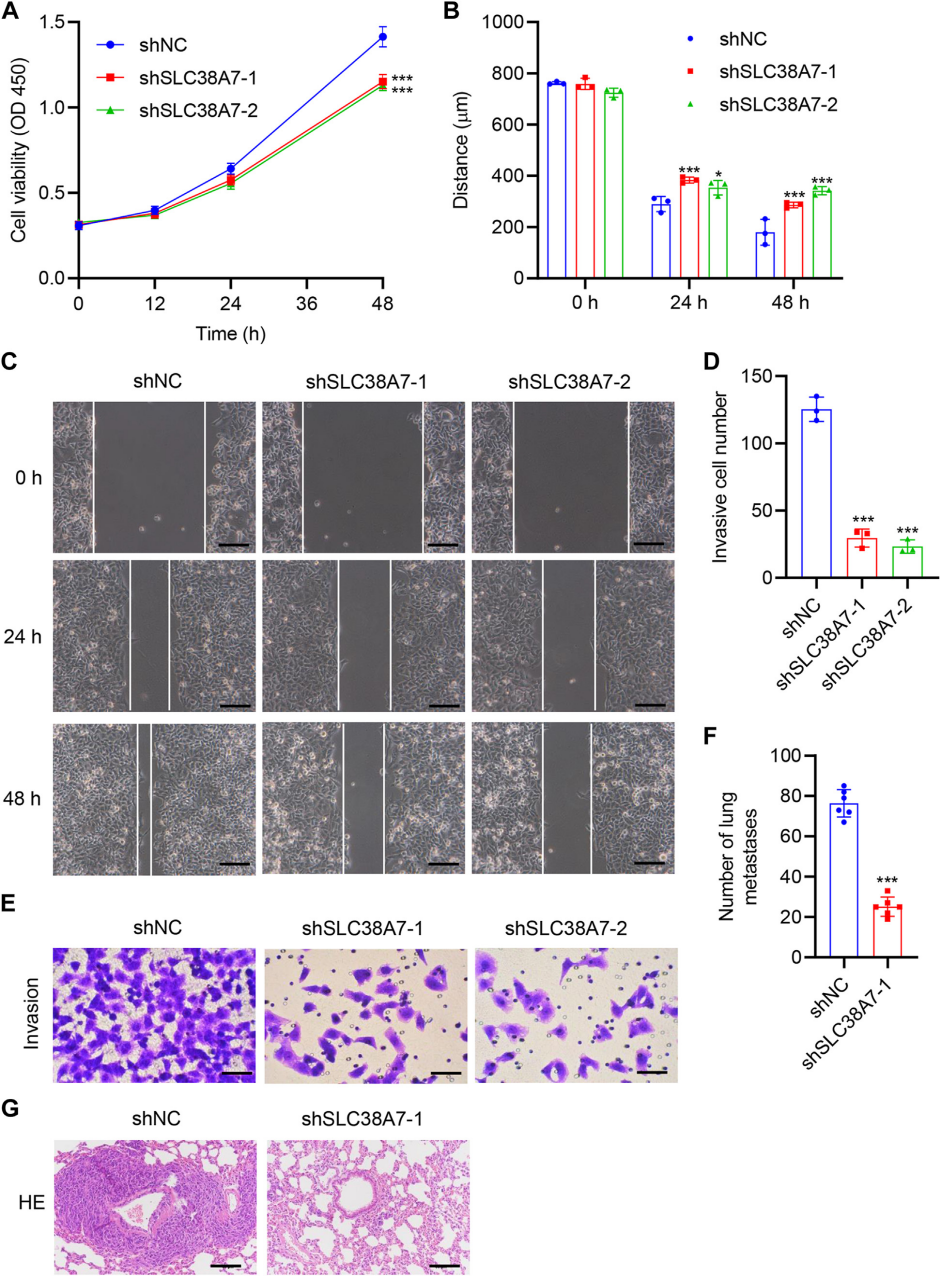
***SLC38A7* knockdown decreases cell viability, migration, and invasion in AGS cells.** To silence *SLC38A7*, AGS cells were transduced with lentivirus-mediated shRNAs targeting *SLC38A7* (shSLC38A7-1, 2). *A*, assessment of cell viability by CCK-8 assay. *B* and *C*, cell migration by wound healing assay (scale bar represents 200 μm). *D* and *E*, cell invasion by Transwell assay (scale bar represents 100 μm). AGS cells transduced with *SLC38A7* shRNA were injected into nude mice through tail veins. *F*, number of lung metastases in mice models. *G*, HE-staining of lung tissue sections (scale bar represents 100 μm). Values are presented as mean ± SD of three or six independent biological experiments. *A*, *B*, and *D*, one-way ANOVA followed by Dunnett’s *post hoc* test or (*F*) unpaired two-tailed student’s *t* test was used. ∗*p* < 0.05, ∗∗∗*p* < 0.001 *vs.* shNC.

To clarify the effect of SLC38A7 on GC cell viability, migration, and invasion, AGS cells were transduced with shSLC38A7-1, 2, and 3 to knock down *SLC38A7*. *SLC38A7* knockdown was confirmed by quantitative RT-PCR (qRT-PCR) and Western blot (Fig. S1*B*). *SLC38A7* knockdown led to significantly decreased cell viability (*p* < 0.001, Fig. 2*A*), migration (*p* < 0.05, Fig. 2, *B* and *C*), and invasion (*p* < 0.001, Fig. 2, *D* and *E*). *In vivo*, we used the tail vein metastasis experiment to confirm this hypothesis and showed that *SLC38A7* knockdown significantly inhibited lung metastasis (*p* < 0.001, Figs. 2, *F* and *G*, S2), consistent with the results *in vitro*.

### *SLC38A7* knockdown inhibited oxidative phosphorylation and induces mitochondrial dysfunction in AGS cells

**Figure 3:**
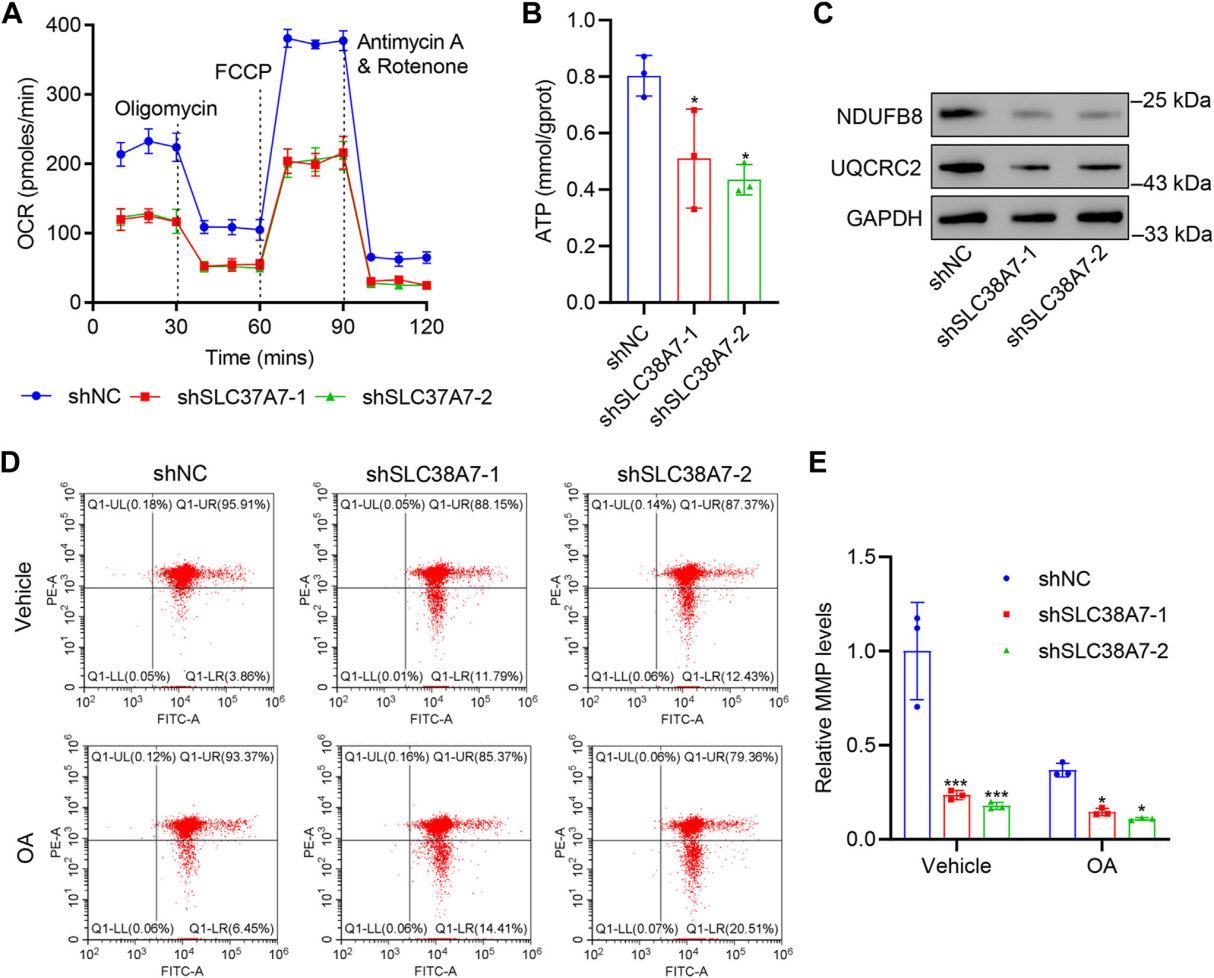
***SLC38A7* knockdown inhibits oxidative phosphorylation and induces mitochondrial dysfunction in AGS cells.** AGS cells were transduced with shSLC38A7-1, 2, or shNC. *A* and *B*, cellular OCR and ATP content. *C*, protein levels of NDUFB8 and UQCRC2. *D* and *E*, mitochondrial membrane potential (MMP) levels detected by flow cytometry. Values are presented as mean ± SD of three independent biological experiments. *B*, and *E*, one-way ANOVA followed by Dunnett’s *post hoc* test was used. ∗*p* < 0.05, ∗∗∗*p* < 0.001 *vs.* shNC.

We further detected the effects of *SLC38A7* silencing on oxidative phosphorylation and mitochondrial function. We observed a significant decrease in OCR values (Fig. 3*A*) and ATP content (*p* < 0.05, Fig. 3*B*) in *SLC38A7*-silenced AGS cells compared to control cells. Ubiquinol-cytochrome c reductase complex core protein 2 (UQCRC2) and NADH dehydrogenase (ubiquinone) 1 beta subcomplex 8 (NDUFB8) are critical proteins in the mitochondrial respiratory chain (14). *SLC38A7* silencing downregulated the levels of UQCRC2 and NDUFB8 proteins in AGS cells (Fig. 3*C*). Consistently, flow cytometry revealed a reduction in mitochondrial membrane potential levels in *SLC38A7*-silenced AGS cells with or without oligomycin and antimycin A (OA) treatment (Fig. 3, *D* and *E*). Collectively, these results demonstrate that *SLC38A7* knockdown suppressed oxidative phosphorylation and induced mitochondrial dysfunction in AGS cells.

### SLC38A7 regulated cell viability, migration, invasion, and oxidative phosphorylation in GC cells

**Figure 4:**
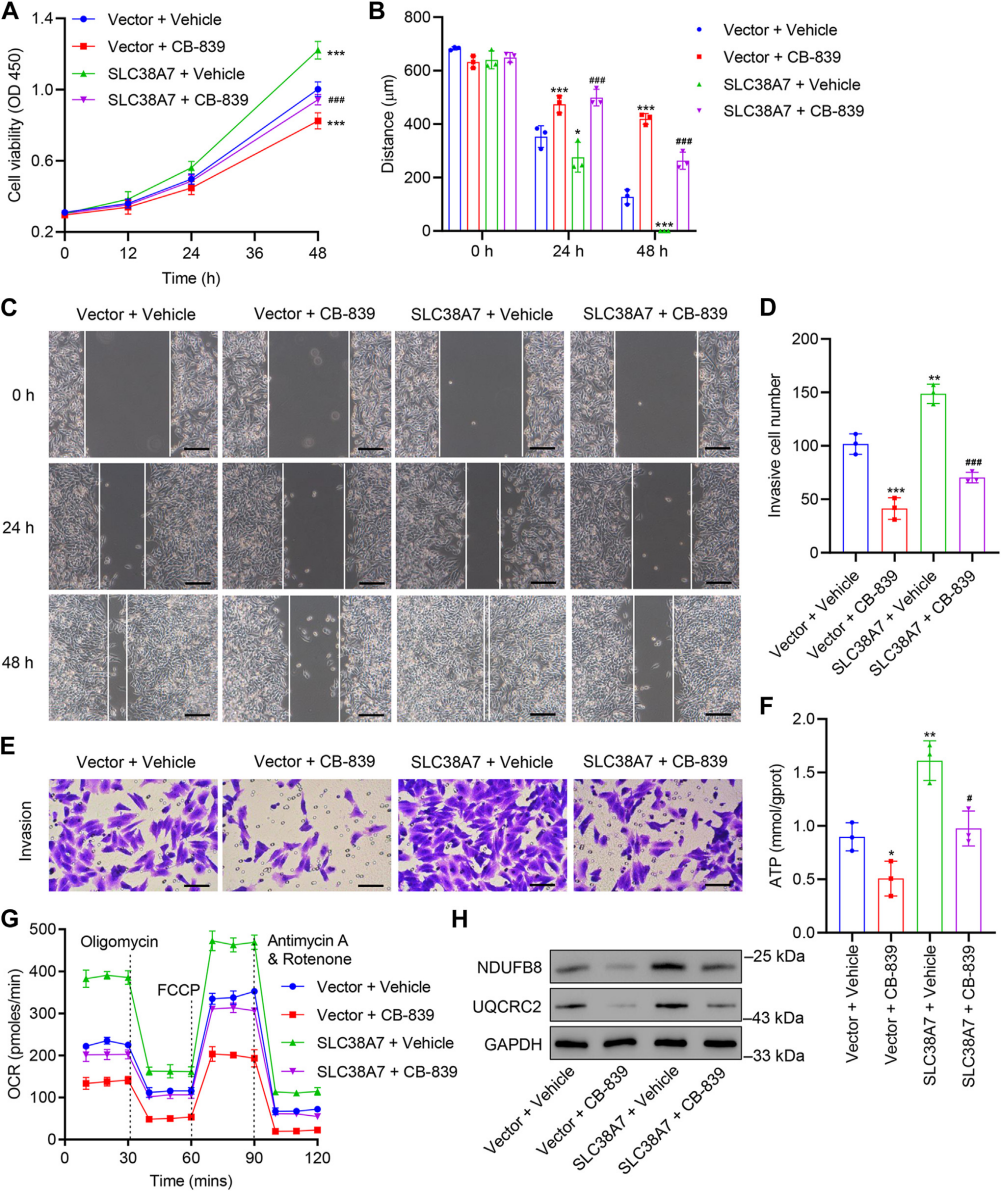
***SLC38A7* overexpression promotes cell viability, migration, invasion, oxidative phosphorylation, and mitochondrial function in HGC27 cells.** HGC27 cells were transduced with *SLC38A7*-overexpressing plasmid or blank plasmid vector prior to CB-839 (glutaminase inhibitor, 10 μM) or vehicle treatment. *A*, assessment of cell viability. *B* and *C*, cell migration by wound healing assay (scale bar represents 200 μm). *D* and *E*, cell invasion by Transwell assay (scale bar represents 100 μm). *F*, ATP content. *G*, cellular OCR value. *H*, protein levels of NDUFB8 and UQCRC2. Values are presented as mean ± SD of three independent biological experiments. *A*, *B*, *D*, and *F*, one-way ANOVA followed by Dunnett’s *post hoc* test was used. ∗*p* < 0.05, ∗∗*p* < 0.01, ∗∗∗*p* < 0.001 *versus* vector + vehicle; ^#^*p* < 0.05, ^###^*p* < 0.001 *vs.* SLC38A7 + vehicle.

To further evaluate the biological role of SLC38A7 and the underlying mechanisms, HGC27 cells were transduced with *SLC38A7*-overexpressing plasmid or blank plasmid vector prior to glutaminase inhibitor CB-839 or vehicle treatment. *SLC38A7* overexpression was confirmed by qRT-PCR and Western blot (Fig. S1*C*). *SLC38A7* overexpression promoted cell viability (*p* < 0.001, Fig. 4*A*), migration (*p* < 0.05, Fig. 4, *B* and *C*), invasion (*p* < 0.01, Fig. 4, *D* and *E*), ATP content (*p* < 0.01, Fig. 4*F*), and OCR values (Fig. 4*G*). However, CB-839 treatment inhibited *SLC38A7* overexpression–induced cell viability, migration, invasion, ATP content, and OCR values (*p* < 0.05, Fig. 4, *A*–*G*). Moreover, the CB-839 treatment alleviated the upregulation in protein levels of UQCRC2 and NDUF8B induced by *SLC38A7* overexpression (Fig. 4*H*).

Moreover, AGS cells were transduced with *SLC38A7* shRNA or shNC prior to CB-839 or vehicle treatment. *SLC38A7* silencing inhibited cell viability (*p* < 0.001, Fig. S3*A*), migration (*p* < 0.001, Fig. S3, *B* and *C*), invasion (*p* < 0.001, Fig. S3, *D* and *E*), and OCR values (Fig. S3*F*). However, CB-839 treatment further promoted *SLC38A7* silencing-induced decreases in the cell viability, migration, invasion, and OCR values (*p* < 0.001, Fig. S3, *A*–*F*).

### METTL3 promoted N6-methyladenosine modification of *SLC38A7* mRNA *via* IGF2BP2

**Figure 5:**
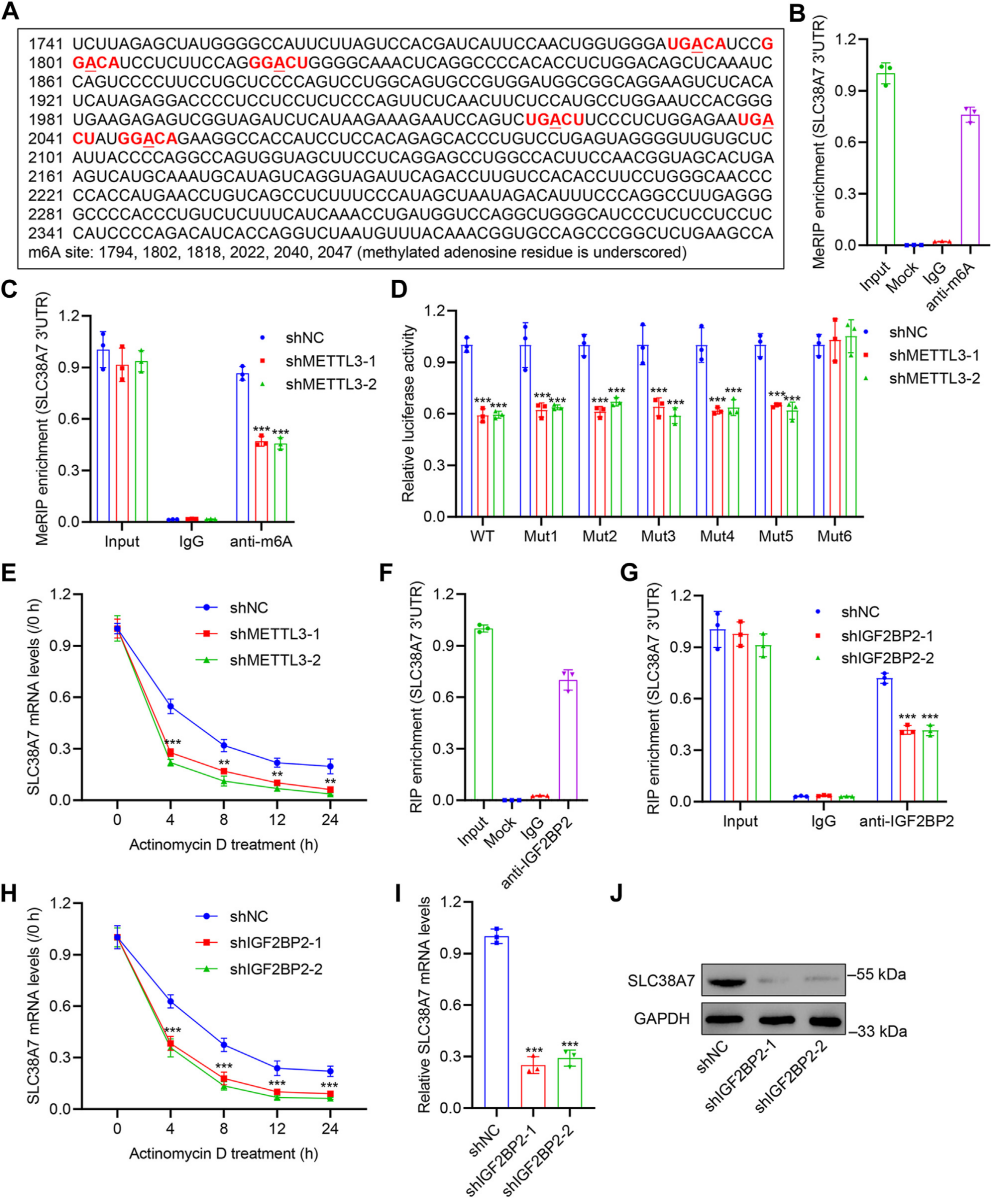
**METTL3 promotes m**^**6**^**A modification of *SLC38A7 via* IGF2BP2.***A*, *SLC38A7* mRNA 3′UTR m^6^A site predicted by sequence-based RNA adenosine methylation site predictor (SRAMP). *B* and *C*, results of MeRIP assay showing the interaction between m^6^A and *SLC38A7* mRNA 3′UTR in AGS cells with or without shNC or shMETTL3 transduction. *D*, detection of relative dual-luciferase activity in AGS cells cotransfected with the *SLC38A7* WT or variants (Mut1-6) luciferase reporter vector, together with the shNC or shMETTL3. *E*, *SLC38A7* mRNA stability in AGS cells transduced with shMETTL3 or shNC. *F* and *G*, results of RIP assay showing the interaction between IGF2BP2 and *SLC38A7* mRNA 3′UTR in AGS cells with or without shNC or shIGF2BP2 transduction. *H*, *SLC38A7* mRNA stability in AGS cells transduced with shIGF2BP2 or shNC. *I* and *J*, SLC38A7 mRNA and protein levels in AGS cells transduced with shIGF2BP2 or shNC. Mock group: Perform IP without adding the antibody. Values are presented as mean ± SD of three independent biological experiments. *C*, *D*, *E*, *G*, *H*, and *I*, one-way ANOVA followed by Dunnett’s *post hoc* test was used. ∗∗*p* < 0.01, ∗∗∗*p* < 0.001 *vs.* shNC.

Previous studies have identified m^6^A modification in RNA as a widespread phenomenon that participates in critical biological functions (15, 16). To illustrate whether *SLC38A7* was regulated by m^6^A modification, sequence-based RNA adenosine methylation site predictor was used and predicted the m^6^A modification sites in the *SLC38A7* mRNA 3′UTR (Fig. 5*A*), and this was confirmed using methylated RNA immunoprecipitation-qPCR (MeRIP-qPCR) (Fig. 5*B*). METTL3 plays a key role in GC *via* m^6^A methylation (17). METTL3 (writer)/IGF2BPs (reader) axis is an important m^6^A-dependent gene regulatory mechanism (18). Correlation analysis of *SLC38A7* and *METTL3* or *IGF2BPs* in the TCGA data using the GEPIA platform is shown in Fig. S4, *A*–*D*. *SLC38A7* expression was positively correlated with *METTL3* and three *IGF2BPs*, with the higher R and lower *p* values of *IGF2BP2* among the three IGF2BPs. METTL3 as well as IGF2BP2 were therefore used for subsequent experiments. We further used MeRIP-qPCR to detect the effect of METTL3 on the m^6^A modification of *SLC38A7* mRNA 3′UTR. *METTL3* knockdown was confirmed by qRT-PCR and Western blot (Fig. S1*D*). The results showed that the knockdown of *METTL3* resulted in a significant decrease in the m^6^A modification of *SLC38A7* mRNA 3′UTR (*p* < 0.001, Fig. 5*C*). Next, we used Luciferase reporters to verify the accuracy of the six predicted sites (A1794, A1802, A1818, A2022, A2040, and A2047) and constructed corresponding *SLC38A7* variants (Mut1-6) by replacing the adenosine in m^6^A consensus sequences with thymine. We found that the mRNA levels of WT *SLC38A7* remarkably decreased after *METTL3* knockdown, but its Mut6 variant was not reduced (Fig. 5*D*), suggesting that the A2047 site in the *SLC38A7* mRNA 3′UTR region is a potential modification site. *METTL3* knockdown also decreased *SLC38A7* mRNA stability (*p* < 0.001, Fig. 5*E*). RNA immunoprecipitation-qPCR (RIP-qPCR) assay demonstrated that 3′UTR of *SLC38A7* mRNA was substantially enriched by IGF2BP2 antibody, providing convincing evidence of their interaction (Fig. 5*F*). Moreover, RIP-qPCR results showed that the knockdown of *IGF2BP2* resulted in a significant decrease in the interaction between IGF2BP2 and *SLC38A7* mRNA 3′UTR (*p* < 0.001, Fig. 5*G*). *IGF2BP2* knockdown was confirmed by qRT-PCR and Western blot (Fig. S1*E*). *IGF2BP2* knockdown also decreased *SLC38A7* mRNA stability (*p* < 0.001, Fig. 5*H*) and downregulated mRNA and protein levels of SLC38A7 (*p* < 0.001, Fig. 5, *I* and *J*). These results collectively demonstrate the involvement of the METTL3–IGF2BP2 axis in regulating SLC38A7 expression *via* an m^6^A methylation-dependent manner. We further evaluated the correlations of protein levels of METTL3 and SLC38A7 in the GC tissue microarrays. IHC staining intensities of METTL3 and SLC38A7 were coincidentally strengthened or alleviated (Fig. S5*A*). SLC38A7 IHC score showed a positive correlation with METTL3 IHC score (*p* < 0.001, Spearman r = 0.544, Fig. S5*B*).

### METTL3/IGF2BP2-mediated m^6^A modification of *SLC38A7* mRNA promoted cell viability, migration, invasion, oxidative phosphorylation, and mitochondrial function in GC cells

**Figure 6:**
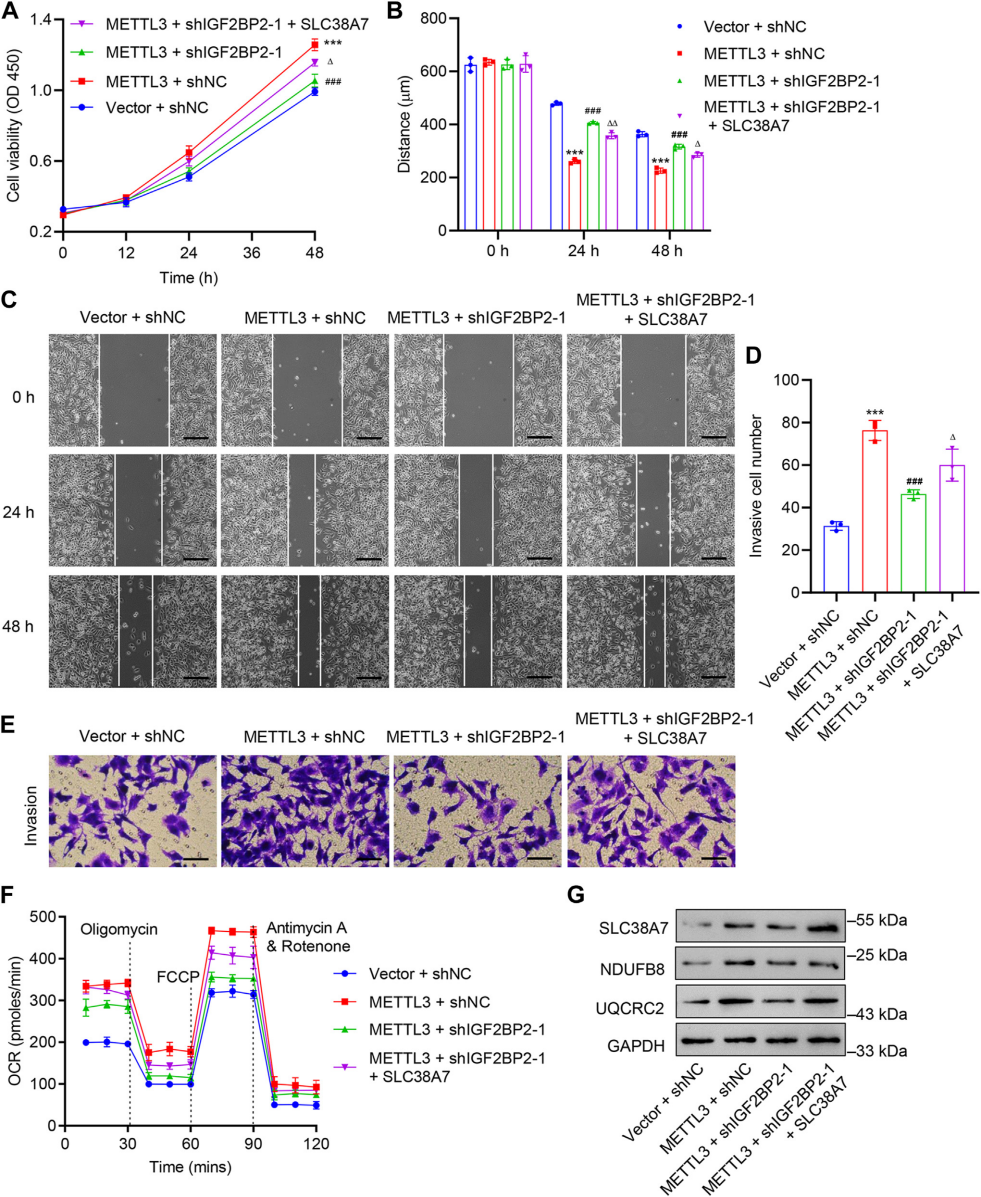
**METTL3/IGF2BP2-mediated m**^**6**^**A modification of *SLC38A7* mRNA promotes cell viability, migration, invasion, oxidative phosphorylation, and mitochondrial function in HGC27 cells.** HGC27 cells were transduced with *METTL3*-overexpressing plasmid, *IGF2BP2* shRNA and *SLC38A7*-overexpressing plasmid. *A*, assessment of cell viability. *B* and *C*, cell migration by wound healing assay (scale bar represents 200 μm). *D* and *E*, cell invasion by Transwell assay (scale bar represents 100 μm). *F*, cellular OCR value. *G*, protein levels of SLC38A7, NDUFB8, and UQCRC2. Values are presented as mean ± SD of three independent biological experiments. *A*, *B*, and *D*, one-way ANOVA followed by Dunnett’s *post hoc* test was used. ∗∗∗*p* < 0.001 *versus* vector + shNC; ^###^*p* < 0.001 *versus* METTL3 + shNC; ^Δ^*P* < 0.05, ^ΔΔ^*P* < 0.01 *versus* METTL3 + shIGF2BP2-1.

To further evaluate the biological role of METTL3/IGF2BP2-mediated m^6^A modification of *SLC38A7* mRNA in GC progression, HGC27 cells were transduced with *METTL3* expression vector or blank vector, along with *IGF2BP2* shRNA and *SLC38A7* expression vector. *METTL3* overexpression was confirmed by qRT-PCR and Western blot (Fig. S1*F*). *METTL3* overexpression led to significantly increased cell viability (*p* < 0.001, Fig. 6*A*), migration (*p* < 0.001, Fig. 6, *B* and *C*), invasion (*p* < 0.001, Fig. 6, *D* and *E*), OCR value (Fig. 6*F*), and protein levels of UQCRC2 and NDUF8B (Fig. 6*G*). However, *IGF2BP2* silencing inhibited *METTL3* overexpression-induced cell viability, migration, invasion, OCR values, and UQCRC2 and NDUF8B expression (*p* < 0.001, Fig. 6, *A*–*G*). Moreover, the *SLC38A7* overexpression could partially reverse *IGF2BP2* knockdown-mediated effects in HGC27 cells (*p* < 0.05, Fig. 6, *A*–*G*).

## Discussion

**Figure 7:**
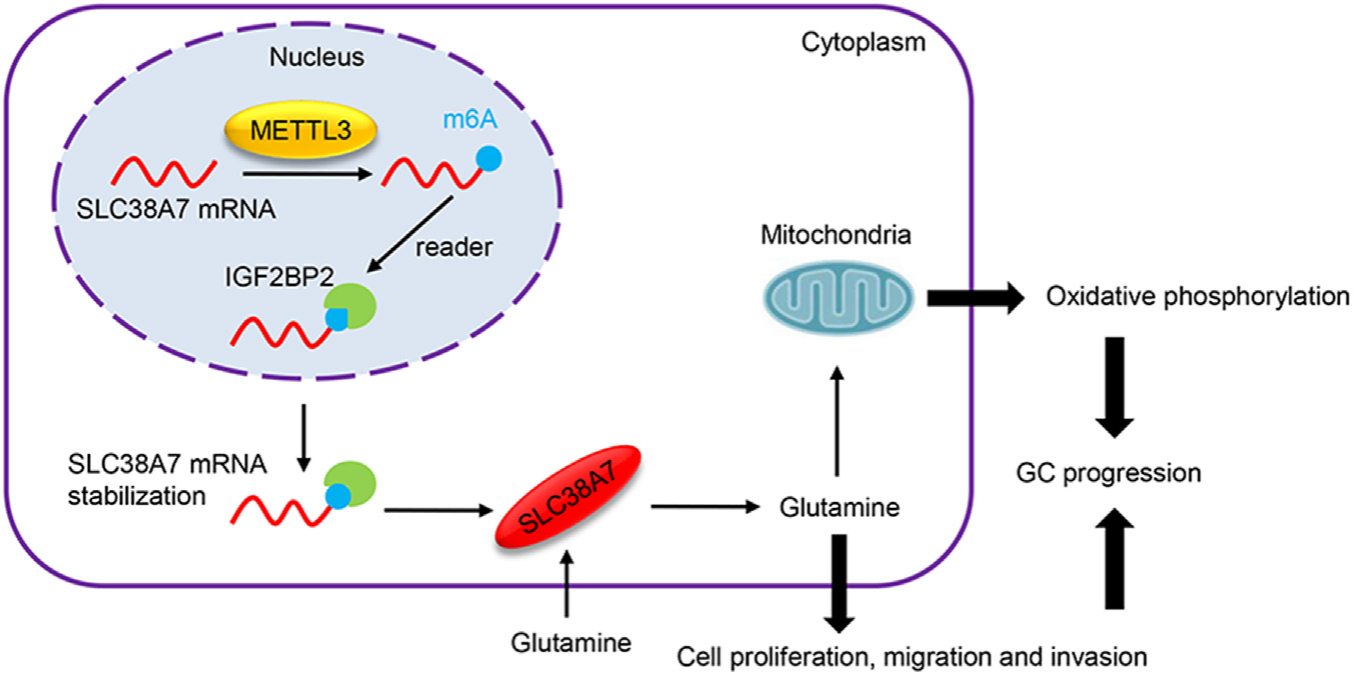
**A graphical summary of the study.** METTL3/IGF2BP2-mediated *SLC38A7* m^6^A RNA methylation exhibits pro-tumorigenic effects in GC.

GC is characterized by high morbidity and mortality rates (19). Tumor cells consume excessive glutamine rapidly to facilitate their proliferation, which produces intermediates and bioenergetics for macro-molecular synthesis (20). SLC superfamily of transmembrane transporters is responsible for the transport of amino acids across membranes, thus participating in various tumorigenic steps, such as proliferation, invasion, and metastasis (21). In the present study, analysis of TCGA data and tumor tissue microarrays revealed upregulation of SLC38A7 in GC. High SLC38A7 expression was associated with shorter survival of GC patients, which is consistent with a previous study on lung squamous cell carcinoma (12). Of note, the present study is the first study to demonstrate the pro-tumorigenic effects of METTL3/IGF2BP2-mediated *SLC38A7* m^6^A methylation in GC (Fig. 7).

Glutamine plays a crucial role in energy regulation and biosynthesis, thereby affecting cancer survival and growth (6, 22). Glutamine serves as an important source of carbon and nitrogen to produce ribose, citrate, nonessential amino acids, and glycerin for promoting the viability and proliferation of cancer cells (23). Four SLC families (including SLC1, SLC6, SLC7, and SLC38) are responsible for the trans-membrane exchange of different substrates including glutamine (24). Glutamine regulates matrix metalloproteinases and plays an essential role in migration and invasion of cancer cells (21, 25). In addition to the role of SLC38A7 in transporting glutamine, it has recently been identified as a poor prognostic marker in small-cell lung cancer and lung squamous cell carcinoma and regulates cell proliferation (12, 26). Moreover, SLC38A7 also regulates pancreatic cancer cell growth through mTORC1 (11), and inactivation of mTORC1 signaling pathway inhibits the growth and metastasis of GC (27). In lines with the previous studies, our study demonstrates for the first time that higher *SLC38A7* expression is associated with a poor survival of GC patients and overexpression of *SLC38A7* strengthens cell viability, migration, invasion, oxidative phosphorylation, and mitochondrial function. Furthermore, in the *in vivo* study, *SLC38A7* deficiency decreased metastatic lung nodules in mice xenograft models. These data suggest that SLC38A7 is a potential novel anticancer target in GC. Given the critical role in tumor progression, inhibiting glutamine intake has emerged as an attractive therapeutic target for GC (28). Therefore, glutamine uptake induced by SLC38A7 would be further confirmed using metabolic tracing. In addition to SLC38A7, SLC38A6 expression was also increased in GC patients but it showed no influence on survival based on the analysis of the GSE62254 database and GC tissue microarray. However, in a previous study, circ_0110940 was found to exert an anti-apoptotic and pro-proliferative effects in GC cells *via* the miR-1178-3p/SLC38A6 axis (29). Despite no significant difference in the expression of other SLC38 family members between GC and normal samples in TCGA data using GEPIA, SLC38A1 and SLC38A2 were correlated with a poor prognosis in GC patients and regulated proliferation, invasion, and migration of GC cells (13, 30). Therefore, further studies are required to examine the role of other SLC38 family members in the development and progression of GC.

As the most abundant methylation modification, m^6^A RNA modification is dysregulated in various tumor processes, such as proliferation and metastasis (31). METTL3 is an m^6^A writer (methyltransferase) responsible for catalyzing m^6^A modification, while IGF2BP2 is an m^6^A reader recognizing target RNA (32). Several lines of evidence support the important roles of METTL3 and IGF2BP2 in GC. For example, Smad3 mRNA could be modified by METTL3-mediated m^6^A, which was subsequently recognized and stabilized by IGF2BP2, thereby enhancing Smad3 protein expression and promoting the activation of TGF-β/Smad pathway in GC to promote proliferation and metastasis (33). SUV39H2 promoted GC proliferation and inhibited apoptosis and chemosensitivity, which was m^6^A modified by METTL3 and stabilized by IGF2BP2 (34). In this study, similarly, we found that METTL3 enhances m^6^A modification of *SLC38A7* transcripts and that IGF2BP2 recognizes the methylated *SLC38A7* to maintain its mRNA stability. Moreover, analysis of clinical data revealed increased METTL3 expression level positively correlated with SLC38A7 expression level in GC. *SLC38A7* and *METTL3* were associated with a poor survival of GC patients in GSE62254 but not in TCGA data using GEPIA. This phenomenon may due to the differences of the sample size and clinicpathological parameters, including patients’ race, age, gender, TNM stage, *Helicobacter pylori* infection status, HER2 status, TP53 mutation status, differentiation degree, histological subtypes, and therapeutic strategy, between GSE62254 and TCGA data (35). In addition, it is worth mentioning that whether *SLC38A7* or *METTL3* is an independent poor prognostic factor deserves further examination. Recent studies have identified METTL3 as an oncogene in GC, promoting proliferation, migration, invasion, and metastasis of cancer cells (36, 37). METTL3 inhibition has garnered enormous interest as an effective antitumor therapy (38). Previous studies have also reported oncogenic actions of IGF2BP2 (39, 40). Our study further confirms the oncogenic role of METTL3 in GC and indicates that METTL3 promotes the progression of GC through recruiting IGF2BP2 to stabilize *SLC38A7* mRNA stability.

In conclusion, SLC38A7 promotes cell viability, migration, invasion, oxidative phosphorylation, and mitochondrial function in GC. *SLC38A7* mRNA is upregulated by METTL3-mediated m^6^A modification and stabilized by IGF2BP2. Our study improves our understanding of the effects of SLC38A7 in GC and highlights the potential application of the METTL3/IGF2BP2/SLC38A7 axis in developing novel antitumor therapeutic strategies. Further *in vivo* experiments and clinical studies are required to validate these results.

## Experimental procedures

### Ethics approval

The animal studies were approved by the Animal Care and Use Committee of The Affiliated Changzhou No.2 People’s Hospital of Nanjing Medical University.

### Data source and bioinformatics analysis

Expression of SLC38 family members in GC samples was evaluated using analysis tools of GEPIA with TCGA database (41). The prognostic value of *SLC38A6* and *SLC38A7* was assessed using the GSE62254 database and evaluated using the Kaplan–Meier plotter. The gene set enrichment analysis algorithm was used to explore the potential biological function of *SLC38A7* in GC.

### Clinical specimens

GC tissue microarrays of and normal gastric tissue microarrays were purchased from Shanghai Outdo Biotech. Patients who had received any treatment or biological medication before sampling were excluded from the study.

### Immunohistochemistry

The anti-SLC38A6 antibody (ab121572; Abcam), anti-SLC38A7 antibody (PA5-59723; Invitrogen), and anti-METTL3 antibody (15073-1-AP; Proteintech) were applied to the paraffin-embedded sections followed by the secondary antibody (D-3004, Shanghai Long Island Biotec. Co. Ltd). IHC staining of SLC38A6, SLC38A7, and MELLT3 were independently scored by two investigators as previously reported (42). A composite score was calculated by multiplying the staining intensity (0, negative; 1, weak; 2, moderate; 3, strong) and percentage of positive cells (0, <5%; 1, 5%–25%; 2, 25%–50%; 3, 50%–75%; 4, >75%), thus giving a range from 0 to 12.

### Cell culture and transfection

Normal human gastric epithelium (GES-1) and human GC cell lines (AGS, NCIN87, MKN45, and HGC27) were purchased from the Shanghai Institute of Cell Biology. All cells were authenticated by short tandem repeats profiling and cultured for fewer than 6 months after the test. Cells were cultured at 95% humidity, 5% CO_2_, and 37 °C in Dulbecco’s modified Eagle’s medium with 10% fetal bovine serum (Gibco) and 1.0% penicillin-streptomycin (Solarbio) solutions.

The shRNAs against human *SLC38A7*, *MELLT3*, or *IGF2BP2* and scramble shRNA (See Table S1 for sequences) acting as negative control (shNC) were cloned into the pLKO.1 vector (Addgene). The cDNA encoding *SLC38A7* or *METTL3* was cloned into the pLVX-Puro vector (Clontech) to obtain the vector expressing pLVX-Puro-SLC38A7 or pLVX-Puro-METTL3 and the empty vector acted as negative control. The 293 T cells (ATCC) were transfected with the constructs by using Lipofectamine 2000 reagent (Invitrogen) as well. After 48 h of transfection, the recombinant lentivirus vector was collected and transduced into GC cells.

### Cell counting Kit-8 assay

Cell Counting Kit-8 assay was used to measure cell viability. Approximately 3 × 10^3^ cells were cultured in each well of a 96-well plate for 12 h. At 0, 12, 24, and 48 h, 10 μl cell counting kit-8 assay was mixed with the cells in each individual well, and the cells were subsequently cultured for 1 h. Then, the absorbance of each well was measured at 450 nm.

### Wound healing assay

The migration ability of GC cells was evaluated using wound healing assay (43). Briefly, GC cells were seeded in a culture dish (35-mm, 8 × 10^5^ cells/dish). When cells were fully confluent, wounds were created using a sterile pipette tip. The cells were then cultured in a serum-free medium. Distance was calculated based on images obtained at 0, 24, and 48 h.

### Transwell invasion assay

Cell invasion was examined using Transwell chambers as previously reported (44). Briefly, 5 × 10^4^ GC cells suspended in serum-free culture medium were inoculated in upper chamber coated with Matrigel (BD Biosciences). After 24 h, the migrated cells on the lower chamber were fixed with 4% paraformaldehyde, stained with crystal violet, and imaged under a microscope.

### Flow cytometry

Mitochondrial membrane potential ratio was expressed as JC-1 aggregates fluorescence intensity (red)/JC-1 monomers fluorescence intensity (green) by using a JC-1 assay kit (C2006, Beyotime Institute of Biotechnology). Flow cytometry was conducted on CytoFLEX flow cytometry (BD Biosciences).

### Extracellular flux analysis

As previously described, the OCR was estimated using the Seahorse XF24 Extracellular Flux Analyzer (45). Briefly, cells were seeded in XF-24 culture plates and cultured in an incubator (5% CO_2_) for 24 h. Approximately 1 h prior to detection, cells were cultured in XF base medium (Agilent Technologies) in an incubator without CO_2_. Subsequently, oligomycin, FCCP, and antimycin A, and rotenone were injected sequentially.

### Measurement of ATP

ATP content was measured using the ATP assay kit (Nanjing Jiancheng Bioengineering Institute). In brief, the supernatant of cell lysates was mixed with a detection solution. The corresponding total protein amounts were used to normalize ATP concentration.

### Quantitative RT-PCR

Total RNA was extracted using Trizol reagents. On the QuantStudio 5 system, qRT-PCR was performed using SYBR Green kit (ABI). The primer sequences used are listed in Table S2.

### Western blot

The total protein was separated by SDS-PAGE and transferred to PVDF membranes. The membranes were incubated with primary antibodies against SLC38A7 (PA5-144828; Invitrogen), NDUFB8 (ab110242; Abcam), UQCRC2 (ab203832; Abcam), METTL3 (ab195352; Abcam), IGF2BP2 (ab129071; Abcam), and GAPDH (5174; Cell Signaling Technology). Following the incubation with the primary antibody, membranes underwent a triple wash procedure using Tris-buffered saline with Tween 20 and incubated with horseradish peroxidase-conjugated secondary antibodies (ZB-2301, ZB-2305; ZSGB-BIO). Subsequently, the membranes underwent additional Tris-buffered saline with Tween 20 washes, and the signals were visualized using enhanced chemiluminescence substrate.

### *In vivo* tumor xenograft model

The animal studies were approved by the Animal Care and Use Committee of The Affiliated Changzhou No.2 People’s Hospital of Nanjing Medical University. A total of 5 × 10^7^ AGS cells with or without transduction of SLC38A7 shRNAs lentivirus were inoculated into nude mice through tail veins (4–6 weeks) (n = 6 each group). After 6 weeks, mice were sacrificed, and the lungs were fixed in Bouins’ solution. Surface metastatic lesions were counted on all lungs in Bouins’ fixative using a magnifying glass as previously described (46). The lungs were also harvested and processed for H&E staining.

### Methylated RNA immunoprecipitation-qPCR

MeRIP assay was performed using a Magna MeRIP m^6^A Kit (Millipore Sigma). For immunoprecipitation, the RNA fragments were incubated with anti-m^6^A (ab208577) or anti-IgG antibody (ab172730, all from Abcam), after which m^6^A methylation enrichment of mRNA was analyzed using qRT-PCR.

### RNA immunoprecipitation-qPCR (RIP-qPCR) assay

RIP assay was performed using a Magna RIP RNA-Binding Protein Immunoprecipitation kit (Millipore Sigma) (47). Cells were extracted by RIP lysis buffer, followed by incubation with anti-IGF2BP2 (ab128175) or anti-IgG antibody (ab172730, all from Abcam). The co-precipitated RNAs were detected by qRT-PCR.

### Dual-luciferase reporter assay

The fragments of *SLC38A7* containing the WT m^6^A motifs as well as mutant (Mut) m^6^A motifs (m^6^A was replaced by T) were synthesized at Generay Technologies and subcloned into the pGL3-basic dual-luciferase reporter vector (Hanbio). And the plasmids were cotransfected into AGS cells with shMETTL3-1, shMETTL3-2, or shNC. After 48 h, the dual-luciferase reporter assay system was used to measure relative luciferase activity.

### Estimation of mRNA stability

*SLC38A7* mRNA stability was measured by actinomycin D assay. Treatment with actinomycin D (0.2 mM, GlpBio) lasted for 0, 4, 8, 12 and 24 h. The samples were then collected, total RNA was extracted, and cDNA was synthesized. *SLC38A7* mRNA levels were quantified using qRT-PCR.

### Statistical analysis

For GEPIA, considering the different stratifications of sex, age, ethnicity in tumor and normal samples, four-way ANOVA, using sex, age, ethnicity and disease state (Tumor or Normal) as variables for calculating differential expression, was applied. Quantitative data are presented as the mean ± SD of three or six independent biological replicates. Data analyses and visualization were conducted using GraphPad Prism 8.4.2. Owing to the normal distribution of variables assessed using the Shapiro–Wilk test, comparisons between groups were conducted using one-way ANOVA followed by Dunnett’s *post hoc* test or unpaired two-tailed student’s *t* test. Kaplan–Meier plotter with auto select best cutoff (all possible cutoff values between the lower and upper quartiles were computed, and the best-performing threshold was used as a cutoff) and a log-rank test were used for survival analysis. *p* < 0.05 was considered indicative of statistical significance.

## Ethics approval

The animal studies were approved by the Animal Care and Use Committee of The Affiliated Changzhou No.2 People’s Hospital of Nanjing Medical University.

## Data availability

The data used to support the findings of this study are included within the article.

## Supporting information

This article contains supporting information.

## Conflict of interest

The authors declare that they have no conflicts of interests with the contents of this article.
