# Supplementary material for: N6-methyladenosine modification of SLC38A7 promotes cell migration, invasion, oxidative phosphorylation, and mitochondrial function in gastric cancer
Source: J Biol Chem. 2024 Sep 30;300(11):107843. doi: 10.1016/j.jbc.2024.107843 (PMC11555334; doi:10.1016/j.jbc.2024.107843)
Supplement: Supplemental Information [file mmc1.docx]

**Supplemental Table 1. The sequences of shRNAs used in the study**

| **Name** | **Sequences** |
| --- | --- |
| shNC | GGACGAGCTGTACAAGTAA |
| shSLC38A7-1 | GGAGATTGGTTTCCAGAAA |
| shSLC38A7-2 | GGTGGGTGCTGGTCAGCTA |
| shSLC38A7-3 | GACCAGCAGGACAAGATTA |
| shIGF2BP1-1 | CGAAGAGATTCCTCTGAAA |
| shIGF2BP1-2 | GGGTAGATATCCATAGAAA |
| shMETTL3-1 | GGTTGCACGGTTCAAGCAA |
| shMETTL3-2 | CAGTGGATCTGTTGTGATA |

**Supplemental Table 2. The sequences of primer sequences used in the study**

| Name | Sequences |
| --- | --- |
| SLC38A7-F | 5ʹ-GATGCATGCTCACGTGTCTCC-3ʹ |
| SLC38A7-R | 5ʹ-AAGACTTTGCCTAAAATTTGCTTGG-3ʹ |
| METTL3-F | 5ʹ-AACTGCAACGCATCATTCGG-3ʹ |
| METTL3-R | 5ʹ-CCTTTGACACCAACCAAGCA-3ʹ |
| IGF2BP2-F | 5ʹ-ACTGCAGGCTAAGGGAGAGA-3ʹ |
| IGF2BP2-R | 5ʹ-GTCCATCCAACACCTCCCAC-3ʹ |
| GAPDH-F | 5ʹ-AATCCCATCACCATCTTC-3ʹ |
| GAPDH-R | 5ʹ-AGGCTGTTGTCATACTTC-3ʹ |


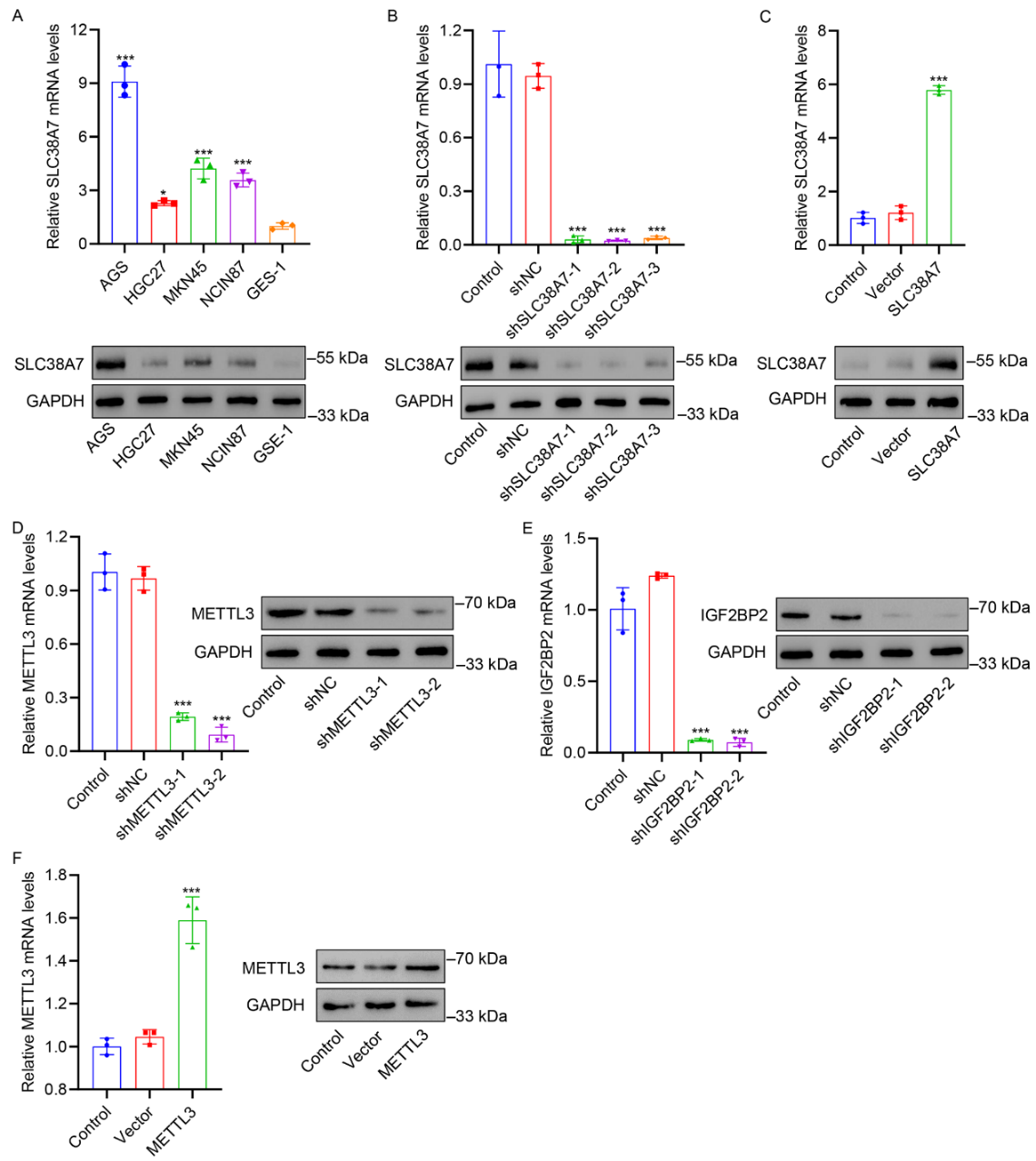


Supplemental Figure 1. Knockdown or overexpression of *SLC38A7*, *METTL3* and *IGF2BP2* in GC cells. (A) mRNA and protein levels of SLC38A7 in various GC cells and normal human gastric epithelium. (B) mRNA and protein levels of SLC38A7 in AGS cells transduced with shSLC38A7-1, 2, 3 or shNC. (C) mRNA and protein levels of SLC38A7 in HGC27 cells with *SLC38A7* overexpression. (D) mRNA and protein levels of METTL3 in AGS cells transduced with shMETTL3-1, 2 or shNC. (E) mRNA and protein levels of IGF2BP2 in AGS cells transduced with shIGF2BP2-1, 2 or shNC. (F) mRNA and protein levels of METTL3 in HGC27 cells with *METTL3* overexpression. Values are presented as mean ± SD of three independent biological experiments. One-way ANOVA followed by Dunnett’s post hoc test was used. **P*<0.05, ****P*<0.001 *vs.* GES-1, shNC or vector.


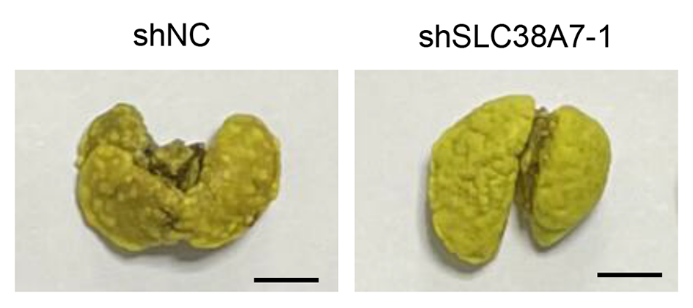


Supplemental Figure 2. *SLC38A7* knockdown inhibits tumor lung metastasis *in vivo*. AGS cells transduced with *SLC38A7* shRNA were injected into nude mice through tail veins. Lung from nude mice were obtained and analyzed (scale bar, 0.5 cm).


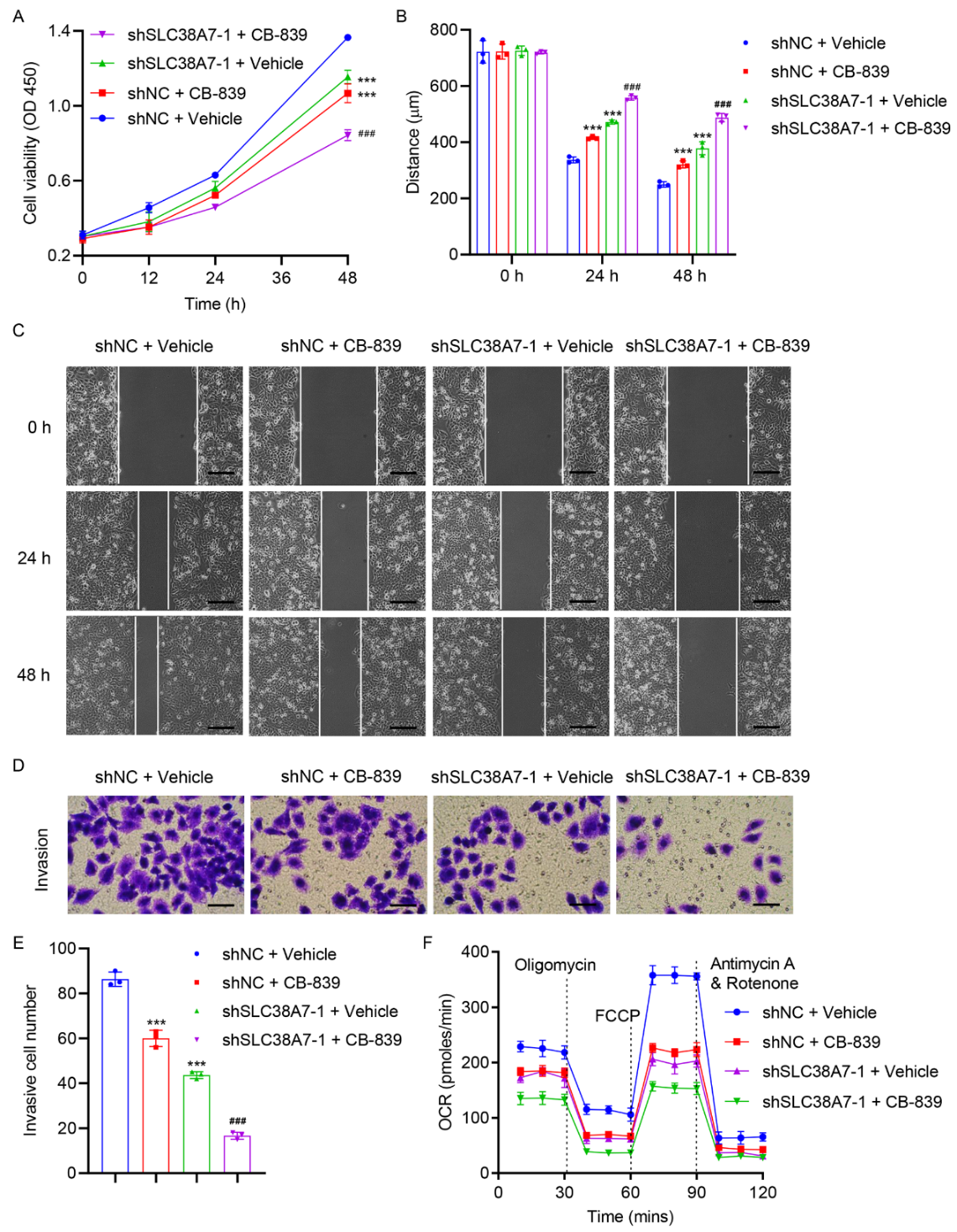


Supplemental Figure 3. Effect of CB-839 on SLC38A7 silencing-induced cell viability, migration, invasion, and oxidative phosphorylation in AGS cells. AGS cells were transduced with *SLC38A7* shRNA or shNC prior to CB-839 (glutaminase inhibitor, 10 μM) or vehicle treatment. (A) Assessment of cell viability. (B, C) Cell migration by wound healing assay (scale bar, 200 μm). (D, E) Cell invasion by Transwell assay (scale bar, 100 μm). (F) Cellular OCR value. Values are presented as mean ± SD of three independent biological experiments. (A, B, E) One-way ANOVA followed by Dunnett’s post hoc test was used. ****P* < 0.001 *vs.* shNC + vehicle; ^###^*P* < 0.001 *vs.* shSLC38A7-1 + vehicle.


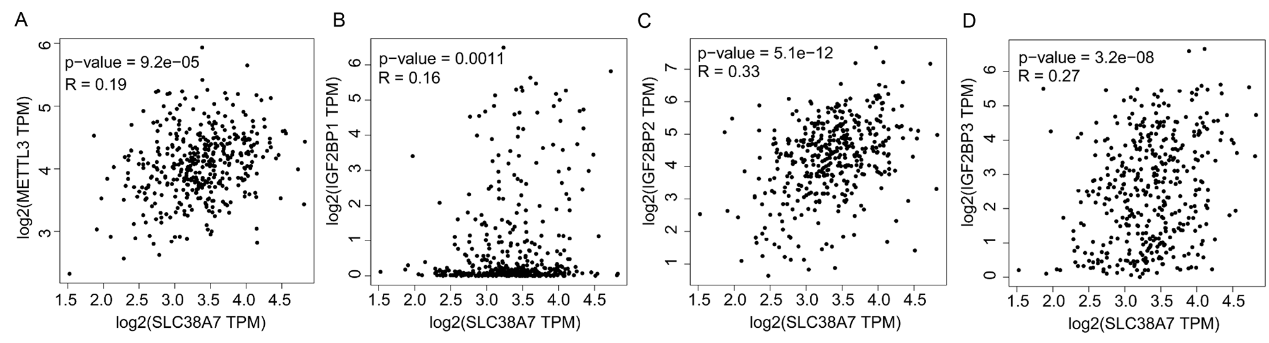


Supplemental Figure 4. (A–D) Correlation analysis of *SLC38A7* and *METTL3*, *IGF2BP1*, *IGF2BP2*, or *IGF2BP3* in GC from TCGA using GEPIA.


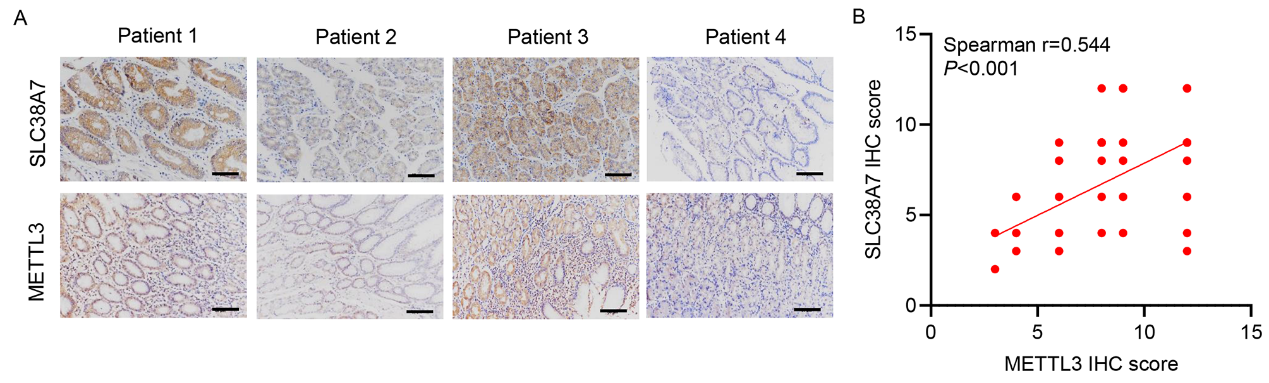


Supplemental Figure 5. Clinical relevance of METTL3 and SLC38A7 levels in GC patients. (A) IHC staining of METTL3 and SLC38A7 in GC tissue microarray (scale bar, 100 μm). (B) Correlation between METTL3 and SLC38A7 IHC scores in GC tissue microarray.
